# Blockade ofthe negative co-stimulatory molecules PD-1 and CTLA-4 improves survival in primary and secondary fungal sepsis

**DOI:** 10.1186/cc12711

**Published:** 2013-05-11

**Authors:** Katherine C Chang, Carey-Ann Burnham, Stephanie M Compton, David P Rasche, RichardJ Mazuski, Jacquelyn SMcDonough, Jacqueline Unsinger, Alan J Korman, Jonathan M Green, Richard S Hotchkiss

**Affiliations:** 1 Department of Anesthesiology,Washington University School of Medicine, 660 South Euclid Ave., St. Louis, MO 63110, USA; 2 Department of Immunology and Pathology,Washington University School of Medicine, 660 South Euclid Ave., St. Louis, MO 63110, USA; 3 Bristol-Myers Squibb, 700 Bay Road, Redwood City, CA 94063, USA; 4 Department of Medicine, Washington University School of Medicine, 660 South Euclid Ave., St. Louis, MO 63110, USA

## Abstract

**Introduction:**

Fungal sepsis is an increasingly common problem in intensive care unit patients.Mortality from fungal sepsis remains high despite antimicrobial therapy that is highly active against most fungal pathogens, a finding consistent with defective host immunity that is present in many patients with disseminated fungemia.One recently recognized immunologic defect that occurs in patients with sepsis is T cell "exhaustion" due to increased expression of programmed cell death -1 (PD-1).This study tested the ability of anti-PD-1 and anti-programmed cell death ligand -1 (anti-PD-L1) antagonistic antibodies to improve survival and reverse sepsis-induced immunosuppression in two mouse models of fungal sepsis.

**Methods:**

Fungal sepsis was induced in mice using two different models of infection, that is, primary fungal sepsis and secondary fungal sepsis occurring after sub-lethal cecal ligation and puncture (CLP).Anti-PD-1 and anti-PD-L1 were administered 24 to 48 h after fungal infection and effects on survival, interferon gamma production, and MHC II expression were examined.

**Results:**

Anti-PD-1 and anti-PD-L1 antibodies were highly effective at improving survival in primary and secondary fungal sepsis.Both antibodies reversed sepsis-induced suppression of interferon gamma and increased expression of MHC II on antigen presenting cells.Blockade of cytotoxic T-lymphocyte antigen-4 (CTLA-4), a second negative co-stimulatory molecule that is up-regulated in sepsis and acts like PD-1 to suppress T cell function, also improved survival in fungal sepsis.

**Conclusions:**

Immuno-adjuvant therapy with anti-PD-1, anti-PD-L1 and anti-CTLA-4 antibodies reverse sepsis-induced immunosuppression and improve survival in fungal sepsis.The present results are consistent with previous studies showing that blockade of PD-1 and CTLA-4 improves survival in bacterial sepsis.Thus, immuno-adjuvant therapy represents a novel approach to sepsis and may have broad applicability in the disorder.Given the relative safety of anti-PD-1 antibody in cancer clinical trials to date, therapy with anti-PD-1 in patients with life-threatening sepsis who have demonstrable immunosuppression should be strongly considered.

## Introduction

Sepsis, the host response to severe infection, is the 10th leading cause of death in the United States and the mostcommon cause of mortality in most intensive care units [1,2].Improved treatment protocols have resulted in the majority of patients surviving the initial 72 hours of sepsis onset only to succumb later in the time course of the disease [3].There is increasing recognition that a state of impaired immunity follows the initial hyper-inflammatory phase of sepsis [4-8].During this phase of impaired immunity, patients are more susceptible to secondary nosocomial infections, often with opportunistic organisms that typically infect immunocompromised individuals.One of the most important opportunistic infections in patients in the ICU is *Candida albicans *[9-12].*Candida *infections are currently the third or fourth most common cause of bloodstream infections in many intensive care units.Although excellent antimicrobial therapy against most *Candida *species exists, mortality remains high at approximately 30 to 40% for fungal sepsis [10-12].

The fact that mortality from fungal infections remains high despite the use of antimicrobial agents that are highly active against fungal organisms, suggests that defects in host immunity may contribute to the persistent high mortality.Therefore, methods that improve host immune function may be fundamental to improving survival. In this regard, recent studies suggest that immuno-adjuvant therapy in invasive fungal infections may be a viable strategy [13-15].IL-7, a pleuripotent cytokine that enhances adaptive immunity throughimmunostimulatory effects on CD4 and CD8 T cells, caused an approximately1.7-fold improvement in survival in a murine fungal sepsis model [13].In addition to animal studies, a few clinical studies support the use of immuno-adjuvant therapy in invasive fungal infections [14,15].A randomized trial of interferon gamma (IFN-γ), a potent activator of macrophages and monocytes in HIV patients with cryptococcal meningitis, showed that treatment led to a significantly faster rate of clearing of cerebrospinal fluid, a finding that has been shown to correlate with survival [14].IFN-γ is currently approved for use in patients with chronic granulomatous disease who have invasive fungal infections [15].

Another potential strategy for improving host immunologic defenses that has shown efficacy in various infectious models is the use of agents which up-regulateadaptive immunity by blocking inhibitory receptors expressed on T lymphocytes [16-19].T cell activation is carefully controlled by expression of positive and negative co-stimulatory molecules that prevent excessive T cell function.CD28 is the classic positive co-stimulatory receptor that, acting in conjunction with the T cell receptor (TCR), induces T cells to proliferate and produce cytokines including, for example, IL-2 and IFN-γ that have extensive effects on other cells.To prevent excessive T cell activation, lymphocytes express a number of negative co-stimulatory molecules that suppress and down-regulate their function [18,19].

Programmed cell death-1 (PD-1) is a member of the B7-CD28 superfamily that functions in an inhibitory role.During T cell activation, PD-1 is rapidly induced and expressed on the surface of CD4 and CD8 T cells where it interacts with its ligands PD-L1 and PD-L2 [20-22].PD-L1 is expressed on both hematopoietic and non-hematopoietic cells and its expression is highly up-regulated during inflammatory states [20].Activation of PD-1 by its ligands causes inhibition ofmanyT cell functions, including proliferation, cytotoxic activity and cytokine production.The essential role of PD-1 in regulating immunity is demonstrated in studies showing that PD-1 knockout mice develop autoimmune diseases, including cardiomyopathy and a lupus-like syndrome [16,20,21].Increased T cell expression of PD-1 is known to occur under conditions of chronic antigenic stimulation, such as persistent viral infections, and lead to T cell "exhaustion" [16,17,20].These "exhausted" T cells are non-functional, prone to undergo apoptosis, and are unable to participate in an effective immune response, thereby contributing to the chronic nature of the viral infections.Blockade of PD-1using inhibitory antibodies has been shown to restore T cell function, increase antiviral T cell responses, and reduce viral load in certain infections [18,19].In addition to viral infections, blockade of the PD-1 pathway has improved survival in bacterial infections.Three independent groups demonstrated that blockade of the PD-1 pathway decreased mortality in clinically-relevant animal models of bacterial sepsis [23-25].The potential clinical relevance of these animal studies is highlighted by recent studies showing that PD-1 over-expression on circulating T cells from patients with sepsis correlated with decreased T cell proliferative capacity, increased secondary nosocomial infections and mortality [26].

The purpose of this study was to determine if inhibition of the T cell negative co-stimulatory PD-1:PD-L1 pathwaycould reverse immune dysfunction and improve survival in primary fungal infections and in secondary fungal sepsis occurring after bacterial infection.Both the anti-PD-1 and the anti-PD-L1 antibodysignificantly improved survival in the two different models of fungal sepsis.This improvement in survival was associated with increased production of IFN-γ, a cytokine which is critical in host defenses against fungal organisms, and reversed the fungal-induced depression of HLA-DR expression in monocytes and dendritic cells.Blockade of cytotoxic T-lymphocyte antigen 4 (CTLA-4), a second negative co-stimulatory molecule that suppresses T cell function, also improved survival in primary and secondary fungal sepsis thereby supporting the concept that immuno-adjuvant therapy offers a rational, new approach to treatment of this highly lethal infection.

## Materials and methods

### Mice

Eight-to-ten-week-old male C57BL6 or CD1 mice were purchased from Jackson Laboratory (Bar Harbor, ME, USA) or Charles River Laboratories (Wilmington, MA, USA), respectively.Procedures were approved by the Animal Studies Committee at Washington University School of Medicine.

### Flow cytometry antibodies and reagents

The following fluorescently labeled antibodies used for flow cytometrywere purchased from BD Pharmingen (San Diego,CA, USA): BD Pharmingen: CD4-FITC (Cat. # 553729), CD4-PeCy5 (Cat. # 553050), CD8-FITC (Cat. # 553031), B220-PeCy5 (Cat. # 553091), CD11c-FITC (Cat. # 553801), CD274-Pe (Cat. # 558091), CD152-Pe (Cat. # 553720), IFNγ-Pe (Cat. # 554412), I-A/I-E-Pe (Cat. # 557000), CD16/CD32-Block (Cat. # 553142).

The following fluorescently labeled antibodies used for flow cytometry were purchased from eBioscience (San Diego, CA, USA): CD279-Pe (Cat. # 12-9985-82), CD11α-FITC (Cat. # 11-0111-85), MHC classII-Pe (Cat.# 15-5322-82).

Anti-PD-1, anti-PD-L1 and anti-CTLA4 antibodies that were used for *in vivo *inhibition studies were provided by Bristol-Meyers Squibb (NewYork, NY; USA).The clones for anti-PD-1 and anti-PD-L1 were 4H2 and 14D8, respectively.Two different clones of anti-CTLA-4 were used.Clone G1 and clone G2B were used for single-hit and two-hit models of fungal sepsis, respectively.

### Fungal sepsis models

*Candida albicans*(ATCC MYA-2430)was grown overnight in Difco™Sabouraud dextrose broth medium (Sigma Aldrich, St. Louis, MO;USA).Cells were harvested, washed and suspended in saline to obtain an optical density of either 0.5*A*_600_or 0.3*A*_600 _for single-hit or two-hit fungal sepsis, respectively,as described [13].

#### Two-hit model of cecal ligation and puncture (CLP) followed by Candida albicans

The CLP model was used to induce sub-lethal peritonitis for use in the two-hit model as previously described [13,27].Mice were anesthetized with isoflurane and a midline abdominal incision was performed.The cecum was ligated and punctured twice with a #27 gauge needle.The abdomen was closed in two layers and mice injected subcutaneously with 1 ml of 0.9% normal saline containing Buprenex(PharmaForce, Columbus, OH; USA)(0.05 mg/kg) subcutaneously and allowed to recover.Imipenem(Merck; Whitehouse Station, NJ; USA)(2.5mg/kg) was given subcutaneously 24h post-CLP.Three days post-CLP, surviving mice received 60 μl of the 0.3*A*_600_*Candida *suspension via tail vein injection.Three days post-CLP was selected as the time point to challenge with *Candida *because of previous studies which demonstrated that mice had increased susceptibility to *Candida *(consistent with impaired immunity) at this time point [27].This dose of *Candida *caused <10% mortality in naïve mice and, therefore, the much higher mortality in mice that had undergone CLP prior to the *Candida *challenge highlights the impaired immunity that occurs following CLP.Mice received fluconazole 200 μgintra-peritoneal (i.p.)five days after *Candida*.Mice were treated with anti-PD-1 (200 μgs), anti-PD-L1 (200 μgs) or anti-CTLA-4 (100 μgs) antibodies beginning at days 2 or 4 post *Candida *infection.

#### Primary Candida (single hit) studies

Unanesthetized mice were injected via tail vein with 50 μl of the 0.5*A*_600_*Candida *suspension.Mice were allowed free access to food and water throughout the study.Where specified, the anti-fungal agent fluconazole (200 μg) was administered via i.p. injection.Fluconazole was used in order to show that immunotherapy with anti-PD-1 was effective when added to standard antimicrobial therapy of fungal sepsis.Anti-PD-1 and anti-PD-L1 antibody administration was started two days after the initial *Candida *infection.Anti-CTLA-4 antibody therapy (50 ugs) was initiated at four days post *Candida *infection.The percentage of mice survival at Day 12 was recorded.

### PD-1 expression in splenic immune cells following*Candida *infection

In order to determine the effect of *Candida *on lymphocyte PD-1 expression following single-hit or two-hit fungal infection, mice were injected with *Candida *as previously described and spleens were harvested at multiple time points post-*Candida *infection [13,27].Splenocytes were prepared and underwent immunostainingwith fluorochrome-conjugated antibodies to CD4 T, CD8 T and PD-1.Flow cytometric analysis (50,000 events/sample) was performed on FACScan (Becton Dickinson, San Jose, CA, USA) and Cell Quest software (BD Pharmigen; San Diego, CA; USA) was utilized to analyze the data [13].

### Quantitation of cytokines

Cytokines were quantified using ELISA Duosets from Invitrogen (Camarillo, CA, USA) and R&D Systems (Minneapolis, MN, USA) employing the μQuantScanning Microplate Spectrophotometer (Bio-Tek Instruments,Winooski, VT, USA) as described [13,24,27].

### Determination of intracellular IFN-γ production in CD4 and CD8 T cells

At Day 11, spleens were harvested and splenocyte suspensions prepared as previously described [13].Approximately 10 million splenocytes were plated in sterile wells with 1 ml of complete RPMI 1640 media (Sigma Aldrich, St Louis, MO; USA).Splenocytes were stimulated with anti-CD3 and anti-CD28 and incubated overnight.Approximately 18 h later, a sample of supernatant was obtained and analyzed for secreted cytokines.Next, brefeldin A was added for an additional four hours incubation after which splenocytes were harvested, washed and immunostained for CD4 or CD8.Cells were then fixed,permeabilized and stained for intracellular IFN-γ.The percentage of cells positive for IFN-γ was determined by flow cytometry as previously described [13].

### Statistical analysis

Data were analyzed with the statistical software Prism (GraphPad, San Diego, CA, USA).Data are reported as the mean ± SEM.For comparison of two groups, the Student's *t*-test was employed. One-way ANOVA with Tukey's multiple comparison tests was used to analyze data in which there were more than two groups. For survival studies, a log rank test was used.Significance was reported at *P *<0.05.

## Results

### Anti-PD-1 and anti-PD-L1 improve survival in two-hit fungal sepsis

**Figure 1:**
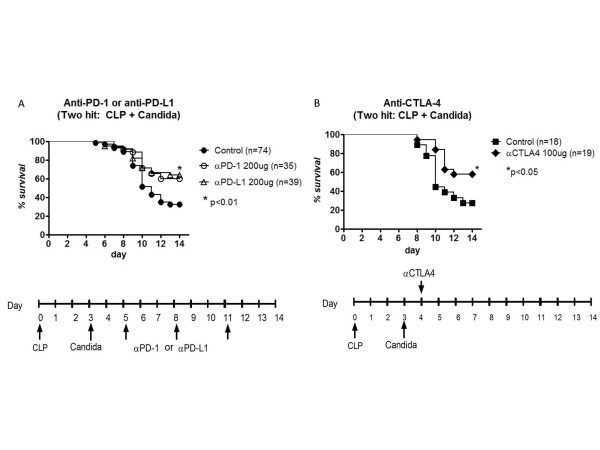
**Anti-PD-1, anti-PD-L1, and anti-CTLA-4 improve survival in two-hit fungal sepsis**. Mice had sub-lethal cecal ligation and puncture (CLP) and three days later had tail vein injection of 60 μl of the 0.3*A*_600_*Candida *suspension.(**A**)Anti-PD-1 or anti-PD-L1 antibody was administered i.p. on days 5, 8 and 11 post-CLP.Fluconazole was administered by i.p. injection daily on days 8 to 12.Survival was recorded for 14 days.Data represent the combined results of four to five individual studies for both anti-PD-1 and anti-PD-L1.Both anti-PD-1 and anti-PD-L1 improved survival compared to control mice treated with saline diluent (*P *<0.01).(**B**) Mice had sub-lethal cecal ligation and puncture (CLP) and three days later had tail vein injection of *Candida*.Anti-CTLA-4 was administered i.p on Day 4 post-CLP. Fluconazole was administered by i.p. injection daily on days 9 to 12.Anti-CTLA-4 improved survival compared to control mice treated with saline diluent (*P *<0.05).Data represent combined results of three studies. CTLA-4, cytotoxic T-lymphocyte antigen-4; PD-1, programmed cell death 1; PD-L1, programmed cell death ligand 1.

Disseminated fungal sepsis frequently occurs as a secondary hospital acquired infection in ICU patients with impaired immunity [9,10].In order to mimic the state of impaired immunity that exists in ICU patients, sub-lethal CLP was performed prior to *Candida *infection as described previously [13,27].Mice underwent CLP followed three days later by *Candida *challenge.Mice were treated by i.p. injection with either anti-PD-1 or anti-PD-L1 antibody or saline diluent (control) starting 48 h after *Candida *infection. Both anti-PD-1 and anti-PD-L1 antibody caused an approximate two-fold improvement in survival in fungal sepsis compared to control (*P *< 0.01)and there was no difference in the two antibody treatments (Figure 1A).Survival studies conducted with isotype control antibodies showed no survival benefit compared to saline control (data not shown).

### Anti-CTLA-4 antibody improves survival in two-hit fungal sepsis

CTLA-4is a negative co-stimulatory molecule that acts in a fashion similar to PD-1 to induce suppression of T cell function [22].Previous work from our group by Inoue *et al*.showed thatanti-CTLA-4 antibody improved survival in a peritonitis model of sepsis and in a two-hit model of CLP followed by *Candida *[28].The study, examining anti-CTLA-4 in the two-hit fungal sepsis model by Inoue *et al.*, was a small preliminary investigation.To further support the concept that inhibition of negative co-stimulatory molecules represents a viable potential therapeutic approach in fungal sepsis, we examined effects of anti-CTLA-4 antibody in a more thorough manner in both primary and secondary (two-hit) models of fungal sepsis.Anti-CTLA-4 antibody was administered 24 h after *Candida *infection and survival was recorded.Anti-CTLA-4 caused a significant improvement in survival compared to mice receiving saline diluent(control), that is, 57.9% versus 27.8%, respectively, *P *< 0.05, (Figure 1B).

### Anti-PD-1, anti-PD-L1, and anti-CTLA-4 improve survival in primary (single-hit) fungal sepsis

**Figure 2:**
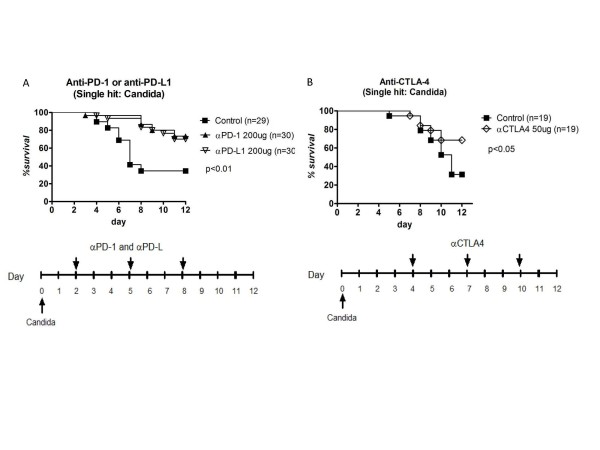
**Anti-PD-1, anti-PD-L1 and anti-CTLA-4 improve survival in single-hit fungal sepsis**. Mice were injected via tail vein with 50 μl of the 0.5*A*_600_*Candida *suspension.(**A**) Anti-PD-1 or anti-PD-L1 antibody was injected i.p. on days 2, 5 and 8 post-*Candida *injection.Fluconazole was administered by i.p. injection daily on days 5 to 10.Mice treated with anti-PD-1 or anti-PD-L1 had significantly improved survival compared to controls (*P *<0.01).Data represent the combined results of three separate studies.(**B**) Anti-CTLA-4 was administered i.p on days 4, 7 and 10 post-*Candida*.Fluconazole was administered by i.p. injection daily on days 10 and 11.Mice treated with anti-CTLA-4 had improved survival versus controls (*P *<0.05).Data represent combined results of two studies. CTLA-4, cytotoxic T-lymphocyte antigen-4; PD-1, programmed cell death 1; PD-L1, programmed cell death ligand 1.

Anti-PD-1, anti-PD-L1 and anti-CTLA-4 were also tested in primary (single-hit) *Candida *infection.Mice had tail vein injection of *Candida *followed by i.p. injections of anti-PD-1 antibody, anti-PD-L1 antibody, or saline diluent at days 2, 5 and 8 following *Candida*.Mice treated with anti-PD-1 or anti-PD-L1 antibody had improved survival compared to control mice (73.3% and 70.0% for anti-PD-1 and anti-PD-L1, respectively, versus 34.5% for controls, *P *<0.01)Figure 2A. Studies of anti-CTLA-4 antibody also demonstrated improved survival versus control mice; the improvement in survivalwas very similar to that occurring with anti-PD-1 and anti-PD-L1 antibody therapy, that is, 68.4% versus 31.5% for anti-CTLA-4 and control respectively, *P *≤0.05, Figure 2B. Studies of isotype control antibody for anti-CTLA-4 showed no difference compared to saline controls (data not shown).

### Lymphocyte PD-1 expression is increased in primary and two-hit fungal sepsis

**Figure 3:**
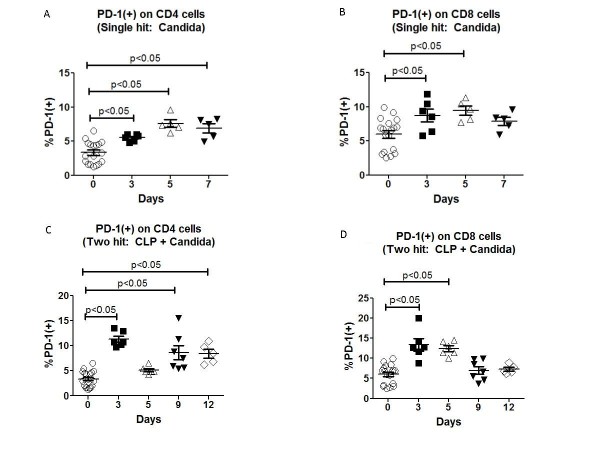
**Single-hit and two-hit *Candida *infection induce increased PD-1 on T cells**. Mice were injected via tail vein with 50 μl of the 0.5*A*_600_*Candida *suspension and spleens harvested on days 0, 3, 5 and 7 post-infections.(**A **and **B**) Expression of PD-1 on CD4 and CD8 T cells was determined by immunostaining and flow cytometry.There was an increase in PD-1 expression on CD4 T cells on all three time points (*P *<0.05); PD-1 expression on CD8 T cells was increased at days 3 and 5 post-infection (*P *<0.05).(**C**and**D**) Mice underwent CLP followed three days later by i.v. injection of *Candida*.Cohorts of mice were killed and spleens harvested on days 3, 5, 9 and 12 post-CLP.PD-1 expression was increased on CD4 and CD8 T cells at Day 3 post-CLP (prior to *Candida *injection) and at days 9 and 12 post-CLP (days 6 and 9 post-*Candida *infection) in CD4 T cells and at Day 5 post-CLP (two days post *Candida *infection) in CD8 T cells (*P *<0.05). CD, cluster of differentiation; CLP, cecal ligation and puncture; PD-1, programmed cell death 1.

We examined expression of PD-1 on CD4 and CD8 T cells following single-hit and two-hit*Candida *infection.Mice were injected with Candidaand spleens were harvested on days 3, 5 and 7 following infection.Splenocyte suspensions were prepared and stained for CD4 or CD8 and PD-1 as described previously [13].There was a significant increase in PD-1 expression on CD4 T cells at all three time points compared to Day 0 (Figure 3A).There wasan increase in PD-1 expression in CD8 T cells at days 3 and 5 following *Candida *infection as well (Figure 3B).

PD-1 expression was quantitated in the two-hit model of sepsis as well.PD-1 expression was increased on CD4 and CD8 T cells at Day 3 following CLP (prior to *Candida *infection).CD4 T cell PD-1 expression was also increased at days 9 and 12 following CLP which corresponds to days 6 and 9 following the second-hit *Candida *infection (Figure 3C).CD8 T cell PD-1 expression was increased at Day 5 post CLP which corresponds to Day 2 following the second-hit *Candida *infection but not at other times (Figure 3D).

### Anti-PD-1, anti-PD-L1 and anti-CTLA-4 increased IFN-γ production in fungal sepsis

**Figure 4:**
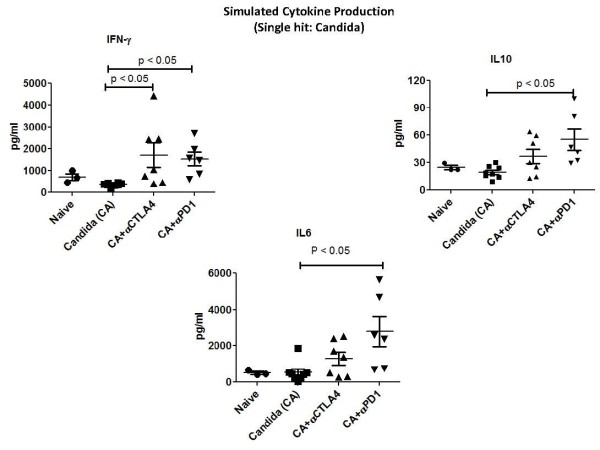
**Anti-PD-1 and anti-CTLA-4 increase splenocyte cytokine production in single-hit fungal sepsis**. Mice had tail vein injection of *Candida *and were treated with anti-PD-1 (days 2, 5 and 8 post-infection) or anti-CTLA-4 (days 4, 7 and 10 post-infection).Mice were killed and spleens harvested on day 12 post-infection.Splenocytes were prepared and stimulated with anti-CD3 and anti-CD28 overnight.Supernatants were harvested and IFN-γ, IL-10 and IL-6 quantitated.Mice treated with anti-PD-1 had increases in all three cytokines compared to *Candida*-infected mice that were treated with saline diluent (controls) (*P *<0.05).Mice treated with anti-CTLA-4 had an increase in IFN-γ only (*P *<0.05). CD, cluster of differentiation; CTLA-4, cytotoxic T-lymphocyte antigen-4; IFN-γ, interferon gamma; IL, interleukin; PD-1, programmed cell death 1.

One potential mechanism for the protective effect of anti-PD-1, anti-PD-L1 and anti-CTLA-4 is to increase IFN-γ, a potent macrophage activator that has shown efficacy in clinical trials of disseminated fungemia [14,15].Thus, the effect of blockade of these inhibitory molecules on IFN-γ production was examined.To study effects in the single-hit model, mice were injected via tail vein with *Candida *and had dosing of anti-PD-1 (days 2, 5 and 8 post-infection) or anti-CTLA4 (days 4, 7 and 10 post-infection).Mice were killed and spleens harvested on Day 12 post-infection.Splenocytes were prepared and stimulated with anti-CD3 and anti-CD28 overnight as described previously.Supernatants were harvested and IFN-γ, IL-10 and IL-6 quantitated.Mice treated with anti-PD-1 had increases in IFN-γ, IL-10 and IL-6 compared to *Candida*-infected mice that were treated with saline diluent (controls) (Figure 4).Mice treated with anti-CTLA-4 had an increase in IFN-γ but not in IL-10 or IL-6 (Figure 4).

**Figure 5:**
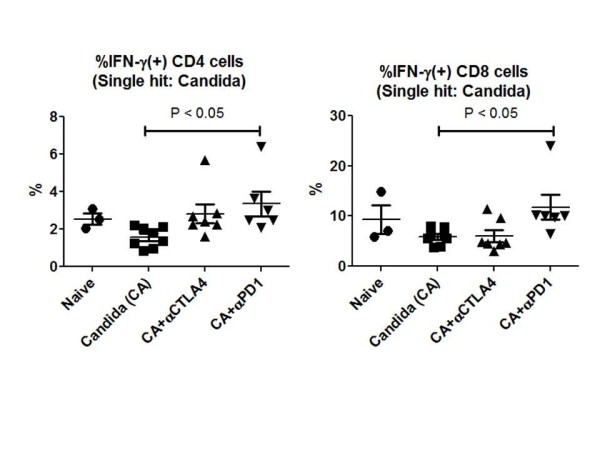
**Anti-PD-1 increases IFN-γ^+ ^CD4 and CD8 T cells in fungal sepsis**. Mice had tail-vein injection of *Candida *and were treated with anti-CTLA-4 or anti-PD-1 as described in Figure 4. Following supernatant harvesting, splenocytes were treated with brefeldin for four hours and then fixed, permeabilized and immunostained for CD4, CD8 and intracellular IFN-γ.Flow cytometry was performed to quantitate the percentage of CD4 and CD8 T cells that were positive for IFN-γ.Anti-PD-1 but not anti-CTLA-4 increased the percentage of IFN-γ^+ ^CD4 and CD8 T cells, (*P *<0.05). CD, cluster of differentiation; CTLA-4, cytotoxic T-lymphocyte antigen-4; IFN-γ, interferon gamma; PD-1, programmed cell death 1.

In addition to quantitating secreted cytokines, the percentage of CD4 and CD8 T cells that were secreting IFN-γ was also quantitated as described previously.Following overnight stimulation with anti-CD3 and anti-CD28, splenocytes were treated with brefeldin for four additional hours. Splenocytes were then fixed, permeabilized and treated with an anti-IFN-γ antibody.The percentage of cells positive for IFN-γ was quantitated by flow cytometry.The percentage of CD4 and CD8 T cells that was positive for IFN-γ was increased in mice treated with anti-PD-1 compared to control mice (*P *<0.05), (Figure 5).

**Figure 6:**
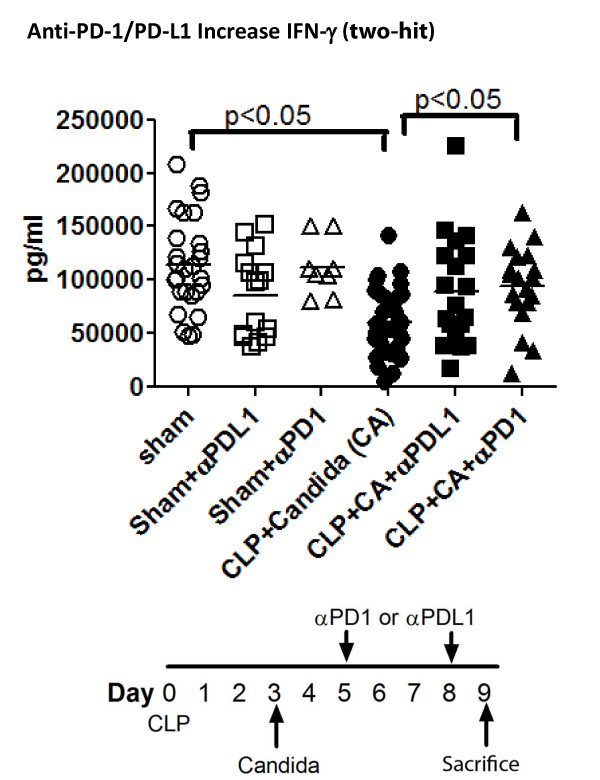
**Anti-PD-L1 increases IFN-γ production in two-hit fungal sepsis**. Mice had CLP followed in three days by *Candida *injection.Anti-PD-1 and anti-PD-L1 were administered on days 5 and 8 post CLP and spleens harvested on Day 9.Splenocyte suspensions were prepared and stimulated overnight with anti-CD3/CD28.Supernatants were harvested at 18 h and IFN-γ production quantitated.Two-hit fungal sepsis caused a decrease in IFN-γ production compared to sham (*P *<0.05); splenocytes from mice treated with anti-PD-1 had an increased IFN-γ production compared to septic mice not receiving antibody therapy.There was a trend toward increased IFN-γ production in mice treated with anti-PD-L1 but it did not reach statistical significance. CD, cluster of differentiation; IFN-γ, interferon gamma; IL, interleukin; PD-1, programmed cell death 1; PD-L1, programmed cell death ligand 1.

A similar series of studies was performed in the two-hit fungal sepsis model.Mice had CLP followed three days later by *Candida*.Mice were treated with anti-PD-1 or anti-PD-L1 on days 5 and 8 post CLP and spleens harvested on Day 9.Splenocyte suspensions were prepared and stimulated with anti-CD3 and anti-CD28 as described.Supernatants were harvested after 18 h and IFN-γ quantitated.Mice treated with anti-PD-1 had a statistically significant increase in IFN-γ compared to control mice (Figure 6); there was also a trend toward an increase in IFN-γ in mice treated with anti-PD-L1.

**Figure 7:**
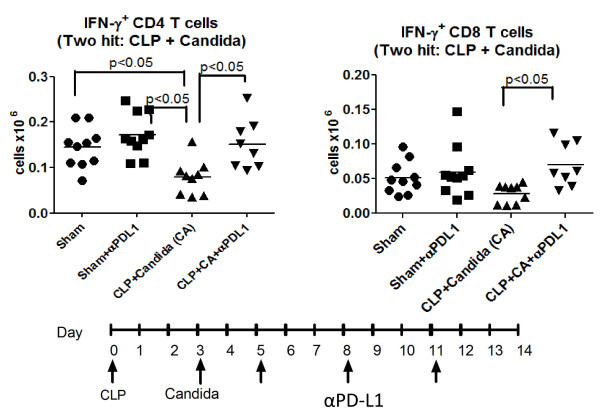
**Anti-PD-L1 increases IFN-γ producing CD4 and CD8 T cells in fungal sepsis**. Mice had CLP followed three days later by *Candida *injection.The protocol for administration of anti-PD-1 and anti-PD-L1 as well as the details of splenocyte preparation and incubation were as described in Figure 6.Following overnight stimulation with anti-CD3/CD28, cells were treated with brefeldin for four hours.Cells were then fixed, permeabilized and stained with anti-IFN-γ.Flow cytometry was performed to quantitate the number of IFN-γ^+ ^CD4 and CD8 T cells. The number of IFN-γ^+ ^CD4 T cells was decreased in two-hit fungal sepsis compared to sham mice and sham mice treated with anti-PD-L1, (*P *<0.05).Septic mice treated with anti-PD-L1 had an increase in the number of IFN-γ^+ ^CD4 and CD8 T cells compared to septic mice treated with saline diluent (*P *<0.05).CD, cluster of differentiation; CLP, cecal ligation and puncture; IFN-γ, interferon gamma; PD-1, programmed cell death 1; PD-L1, programmed cell death ligand 1.

Effects of anti-PD-L1 on intracellular IFN-γ production were quantitated in a separate series of studies.Mice had CLP followed by *Candida *infection three days later. Mice were treated with anti-PD-L1 on days 5, 8 and 11 post CLP and spleens harvested on Day 14.The absolute number of IFN-γ positive CD4 T cells was decreased in mice that had fungal sepsis compared to sham operated mice (Figure 7). Importantly, treatment with anti-PD-L1 restored the percentage of IFN-γ positive CD4 T cells.Anti-PD-L1 treatment also increased the number of splenic CD8 T cells that were producing IFN-γ.

### Anti-PD-1 and anti-PD-L1 increase MHC II expression on antigen presenting cells

**Figure 8:**
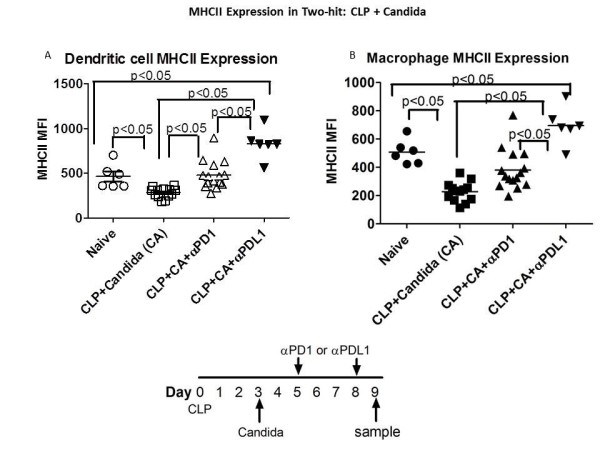
**Anti-PD-1 and anti-PD-L1 increase MHC II expression on dendritic and macrophages in fungal sepsis**. Mice had CLP and three days later were injected with *Candida*.Anti-PD-1 or anti-PD-L1 was administered on days 5 and 8 post-CLP. Spleens were harvested on Day 9 post-CLP and splenocytes prepared.Immunostaining for dendritic cells (CD11c^+^/MCH II hi) and macrophages (F4/80^+^/MHC II intermediate) was performed.The mean fluorescence intensity (MFI) of dendritic cell and macrophage MHC II was decreased in fungal sepsis compared to naïve mice (*P *<0.05).Both anti-PD-1 and anti-PD-L1 increased MHC II MFI in dendritic cells and macrophages in fungal sepsis (*P *<0.05), but anti-PD-L1 had a greater effect compared to anti-PD-1, (*P *<0.05). CLP, cecal ligation and puncture; IFN-γ, interferon gamma; PD-1, programmed cell death 1; PD-L1, programmed cell death ligand 1.

A decrease in macrophage and dendritic cell MHC II expression is a hallmark of sepsis [7].We examined the effects of anti-PD-1 and anti-PD-L1 antibody on MHC II mean fluorescent intensity (MFI) expression on antigen presenting cells in the two-hit model of CLP followed by *Candida *infection.Mice had CLP followed by *Candida *and were treated with anti-PD-1 or anti-PD-L1 at days 5 and 8 post CLP.Spleens were harvested on Day 9 and dendritic cells (CD11c^+^/MHC II hi) and macrophages (F4/80^+^/MHC II intermediate) were identified by immunostaining as described previously [13].The MFI, a measure of the number of MHC II molecules expressed per cell, was quantitated.The decrease in dendritic cell MHC II MFI that occurred with fungal sepsis was reversed by both anti-PD-1 and anti-PD-L1 treatment, (Figure 8). Interestingly, anti-PD-L1 caused a greater increase in MHC II MFI compared to anti-PD-1.Macrophage MHC II MFI was also decreased in fungal sepsis and anti-PD-L1 (but not anti-PD-1) acted to reverse this decrease.

## Discussion

The present results demonstrating that blockade ofPD-1, PD-L1 or CTLA-4 leads to improved survival in both primary and secondary fungal sepsis strongly support the concept that inhibition ofT cell negative co-stimulatory pathways represents an effective therapeutic approach to disseminated fungal sepsis.Previous reports by three independent groups showed that inhibition of the PD-1:PD-L1 pathway improved survival in the clinically relevant CLP model of bacterial sepsis [23-25].Recent studies have also reported that inhibition of PD-1 restores T cell function and decreases viral load in murine models of hepatitis B and HIV-1 [29,30].In a related fashion, our group has reported that an antagonistic antibody to CTLA-4 improved survival in a clinically-relevant animal model of bacterial peritonitis [28].Additional studies havedemonstrated that increased CTLA-4 expression adversely affected pathogen clearance in murine models of *Helicobacterpylori*, *Leishmania *and *Trypanosoma *[31-33].Considered together, these studies make a compelling case that modulation of the negative co-stimulatory pathway mediated by PD-1/PD-L1 and CTLA-4 represents a novel and potentially highly effective approach to a broad range of infectious agents.

The findings from the current studies showing that blockade of negative co-stimulatory pathways improves survival in disseminated fungal sepsisprovide additional support for the hypothesis that immunosuppression is a major pathophysiologic phenomenon in sepsis.Although there are multiple interacting mechanisms responsible for immunosuppression in sepsis, including, for example, increased T regulatory cells, increased myeloid derived suppressor cells, and apoptosis-induced depletion of immune effector cells, recent studies highlight the likely role of PD-1 mediated T cell "exhaustion" in impaired immunity [23-25,34,35].Guignant*et al*. showed that PD-1 over-expression occurred on circulating T cells from patients with sepsis and correlated with decreased T cell proliferative capacity, increased secondary nosocomial infections and mortality [26].Studies from our group documented an increase in the percentage of PD-L1 expressing monocytes in patients who die of sepsis versus sepsis survivors (data not shown).A recent postmortem study from our group also demonstrated significantly increased expression of PD-1 and PD-L1 on splenic T cells and antigen presenting cells respectively in septic versus non-septic patients [34].This increased expression of PD-1 and PD-L1 was associated with marked suppression of stimulated cytokine production by splenocytes from septic patients; splenocytes from septic patients produced less than approximately 10% of the amount of cytokines produced by splenocytes from critically-ill non-septic patients.Collectively, these studies are consistent with a major role for PD-1:PD-L1-mediated immune suppression in patients with sepsis.

Blockade of PD-1, PD-L1 and CTLA-4 not only offers new hope in infectious disease but also appears especially promising in cancer therapy [21,22,36-38].Anti-CTLA-4 antibody reinvigorated the concept of immunotherapy as a valid approach to cancer with its success in inducing significant remissions in patients with widely metastatic malignant melanoma, many of whom had failed other therapies [38].Anti-PD-1 antibody, which has a better safety profile compared to anti-CTLA-4, produced clinical responses in approximately 25% of patients with a variety of diverse tumors [36,37].Anti-CTLA-4 and anti-PD-1 restore T cell function thereby reactivating host immunity allowing it to eliminate tumor cells.Remarkably, many remissions with anti-CTLA-4 and anti-PD-1 appear to be durable, that is, they have lasted for over a year, and may be permanent [39].The encouraging results of cancer immunotherapy with anti-CTLA-4 and anti-PD-1 are highly relevant to sepsis patients because cancer and sepsis share many of the same immunosuppressive mechanisms,including increased T cell CTLA-4 expression and increased PD-1 and PD-L1 expression in T cells and monocytes, respectively, with T cell "exhaustion" [4-8,34,35].Sepsis, like cancer, is an ideal condition for persistent antigenic exposure which is thought to be one mechanism driving induction of PD-1/PD-L1 and T cell dysfunction.

Although there are likely a number of mechanisms that are responsible for the protective effect of anti-PD-1, anti-PD-L1 and anti-CTLA-4 in sepsis, one likely mechanism is their ability to reverse the sepsis-induced suppression in IFN-γ production.All three antibodies had demonstrable efficacy in increasing either splenocyte IFN-γ production or the percentage of IFN-γ positive CD4 and/or CD8 T cells (Figures 4, 5, 6, 7).(Note that differing effects on host immunity have recently been observed in animals treated with different isotypeantibodies to CTLA-4 [40].)T cells are major producers of IFN-γ, which is essential for optimal function of monocytes and macrophages.Sepsis-induced suppression of IFN-γ production is likely a major driving force for the immune suppression in the disorder [41,42].Administration of IFN-γ during the immunosuppressive phase of sepsis has been reported to be beneficial in several small clinical studies [41,42].The ability of anti-PD-1, anti-PD-L1 and anti-CTLA-4 antibodies to improve IFN-γ production may be particularly germane to fungal sepsis.Administration of IFN-γ improves outcome in patients with chronic granulomatous disease who have fungal sepsis.Recently, a trial of IFN-γ in a small number of patients with fungal meningitis showed that IFN-γ leads to more rapid clearing of fungal organisms from the cerebrospinal fluid, an important clinical finding that correlates with survival [14].

Another interesting finding regarding effects of the anti-PD-1 antibody on cytokines was its action to increase production of the pro-inflammatory cytokine IL-6 and the immunosuppressive cytokine IL-10 (Figure 4).The effect of anti-PD-1 to increase IL-10 in the present study is somewhat surprising given findings by Said *et al*. who reported that PD-1 induces IL-10 production in monocytes in patients with HIV, thereby resulting in impaired T cell activation [43].Our findings, showing an effect of anti-PD-1 antibody to increase IL-10 production, are similar to the report by Wong *et al*. who noted that anti-PD-L-1 antibody caused an increase in both IFN-γ and IL-10 in a lymphocyte incubation study [44].The increase in IL-10 splenocyte production in the present study was relatively small compared to the larger effect of the anti-PD-1 antibody on IFN-γ production (Figure 4).Thus, we believe that the predominant effect of the anti-PD-1 antibody is to enhance activation of T cells, a highly beneficial effect in sepsis.

Similar to our previous findings in the CLP model of sepsis [28], the anti-CTLA-4 antibody worsened outcome in fungal sepsis if administered at higher doses or too early during the course of the disease (data not shown).No such adverse effects were observed with anti-PD-1 or anti-PD-L1 antibody at any dose or time of administration (data not shown).Thus, anti-PD-1 and anti-PD-L1 appear to have a better safety profile compared to anti-CTLA-4 antibody and this finding mirrors clinical studies in cancer patients which show a lower incidence of autoimmune effects in patients treated with anti-PD-1 compared to anti-CTLA-4 [36-39].

Additional evidence for beneficial effects of anti-PD-1 and anti-PD-L1 on host immunity is provided by their ability to increase MHC II expression on macrophages and dendritic cells (Figure 8).Decreased MHC II expression is a hallmark of sepsis and is used as a marker of immune suppression during the disorder [7].MHC II molecules function to present microbial antigens to CD4 T cells thereby activating these cells for optimal response to the infectious challenge.Although both anti-PD-1 and anti-PD-L1 improved dendritic cell and macrophage MHC II expression, anti-PD-L1 had a greater effect than anti-PD-1 (Figure 8).This more robust effect of anti-PD-L1 antibody compared to anti-PD-1 antibody to increase MHC II expression may be related to the fact that PD-L1 is expressed on macrophages and dendritic cells and has direct suppressive effects on these cells.

The present experimental findings providepreclinical hypothesis-generating data which support the contention that immuno-adjuvant therapy with anti-PD-1/PD-L1 and anti-CTLA-4 offers a new approach to treatment of fungal infections and likely infectious disease in general.The remarkable efficacy of anti-PD-1 and anti-CTLA-4 antibody therapy to induce remissions in a relatively high percentage of cancer patients [36-38] is evidence that this immunologic approach has profound effects to enhance host immunity.Sepsis, another life threatening failure of host immunity that shares many immune defects with cancer, is likely amenable to this immuno-stimulatory approach as well.Although serious autoimmune side effects of anti-PD-1 and anti-CTLA-4 have occurred in a small percentage of cancer patients treated with these agents [36-38], these adverse effects would likely be less problematic in patients with sepsis because of two factors.First, compared to cancer patients, most septic patients have more severe impairment in host immunity.Secondly,most patients with sepsis would need only short-term therapy with anti-PD-1 or anti-CTLA-4 and, therefore, would be less likely to develop autoimmunity.Additionally, anti-PD-1 antibody could be tailored to septic patients whose peripheral circulating T cells or monocytes have persistently increased expression of PD-1 or PD-L1, respectively, evidence that the patients have entered an immunosuppressive phase of the disorder.Thus, a readily available biomarker could be used to select ideal candidate patients for immune therapy using these agents.

## Conclusions

Blockade of the negative co-stimulatory molecules PD-1, PD-L1 and CTLA-4 improved survival in two models of fungal sepsis.Potential mechanisms of action for the beneficial effects of blocking negative co-stimulatory molecules include increased IFN-γ production and increased expression of MHC II on antigen presenting cells.If ongoing, large phase 3 studies of anti-PD-1 antibody in cancer patientscontinue to show its relative safety, carefully conducted clinical trials of anti-PD-1 antibody should be conducted in patients with sepsis.Such a novel immunologic therapy could represent a major advance against this highly lethal disease.

## Key messages

• Fungal sepsis is an increasingly important cause of morbidity and mortality in intensive care unit patients.

• Programmed cell death-1 (PD-1), programmed cell death ligand-1 (PD-L1) and cytotoxic T-lymphocyte antigen-4 (CTLA-4), are important inhibitors of immune cell function which have be shown to be increased in patients with sepsis.

• Antibodies to PD-1, PD-L1 and CTLA-4 improved survival in two different models of fungal sepsis.

• Anti-PD-1 increased production of IFN-γ, an important activator of monocyte/macrophages, and increased expression of major histocompatibility molecule II.

• Anti-PD-1 and anti-CTLA-4 antibodies may offer a new immunomodulatory therapeutic approach in fungal sepsis.

## Abbreviations

CD: cluster of differentiation; CLP: cecal ligation and puncture; CTLA-4: cytotoxic T-lymphocyte antigen-4; DTH: delayed type hypersensitivity response; FACscan: fluorescent activated cell sorter; HLA-DR: human leukocyte antigen-DR; IFN-γ: interferon gamma; IL-6: interleukin 6; IL-10: interleukin 10; i.p.:intra-peritoneal; MHC II: major histocompatibility complex II; MFI: mean fluorescence intensity; PD-1: programmed cell death 1; PD-L1: programmed cell death ligand 1; TCR: T cell receptor

## Competing interests

Dr. Hotchkiss has received research laboratoryfunding from Bristol Meyers Squibb, Medimmune, Pfizer, Agennix, Aurigene, and by the National Institutes of Health grants GM055194 and GM044118.Dr. Korman is an employee of Bristol-Meyers Squibb.

## Authors' contributions

KCC and JU performed flow cytometry studies. CAB performed microbiologic studies.SMC, DPR and RJM processed tissue samples and helped analyze data.JSM and JU performed animal surgeries.AJK, JMG and RSH helped design experimental studies.RSH wrote the manuscript. All authors read and approved the final manuscript.
